# Preparation of atorvastatin calcium-loaded liposomes using thin-film hydration and coaxial micromixing methods: A comparative study

**DOI:** 10.1016/j.ijpx.2024.100309

**Published:** 2024-11-29

**Authors:** Faezeh Dangkoub, Mehri Bemani Naeini, Shima Akar, Ali Badiee, Mahmoud Reza Jaafari, Mojtaba Sankian, Mohsen Tafaghodi, Seyed Ali Mousavi Shaegh

**Affiliations:** a Department of Pharmaceutical Nanotechnology, School of Pharmacy, Mashhad University of Medical Sciences, Mashhad, Iran; b Nanotechnology Research Center, Pharmaceutical Technology Institute, Mashhad University of Medical Sciences, Mashhad, Iran; c Laboratory of Microfluidics and Medical Microsystems, Research Institute for Medical Sciences, Mashhad University of Medical Sciences, Mashhad, Iran; d Orthopedic Research Center, Mashhad University of Medical Sciences, Mashhad, Iran; e Biotechnology Research Center, Pharmaceutical Technology Institute, Mashhad University of Medical Sciences, Mashhad, Iran; f Immunology Research Center, Mashhad University of Medical Sciences, Mashhad, Iran; g Clinical Research Unit of Ghaem Hospital, Mashhad University of Medical Sciences, Mashhad, Iran

**Keywords:** Nanoliposome, Coaxial micromixer, Thin-film hydration, Microfluidics, Controllability, Reproducibility, Comparison

## Abstract

Development of techniques to produce nanoformulations in a controlled and reproducible manner is of great importance for research, clinical trials, and industrial scale-up. This research aimed to introduce a cost-effective micromixing approach for the nanoassembly of liposomes and compared with thin-film hydration (TFH) method. Numerical simulations and design of experiments (DOE) by response surface methodology (RSM) were used to evaluate the effects of input parameters on liposome properties, aiming to identify optimal conditions. Anionic liposomes without or with atorvastatin calcium (ATC) produced using TFH and the micromixing methods showed similar characteristics in size (150–190 nm), PDI (<0.2), and zeta potential (−50 to −60 mV). Both methods achieved about 70 % encapsulation efficiency with similar drug release profile for ATC-containing liposomes. Analysis of stability and DSC thermograms revealed comparable outcomes for liposomes prepared using both techniques. Nanoliposomes produced via both approaches indicated similar in vitro biological performance regarding cellular uptake and cell viability. The micromixing approach presented an alternative method to produce nanoliposomes in a one-step manner with high controllability and reproducibility without requiring specialized equipment. Compatibility of the micromixer with various solvents, including those detrimental to conventional microfluidic materials like PDMS and thermoplastics, enables exploration of a wide range of formulations.

## Highlights

• A metallic-glass micromixer, resistant to various solvents to produce a wide range of formulations was developed.
• The cost-effective coaxial micromixer produced liposomal nanocarriers (NCs) in a single step using nanoprecipitation method.
• Liposomes made by micromixing method in a continuous manner showed similar results to those produced by TFH approach.
• Compared to TFH, the micromixing method provided high controllability and reproducibility, using a few items of equipment.

## Graphical abstract

**Figure:**
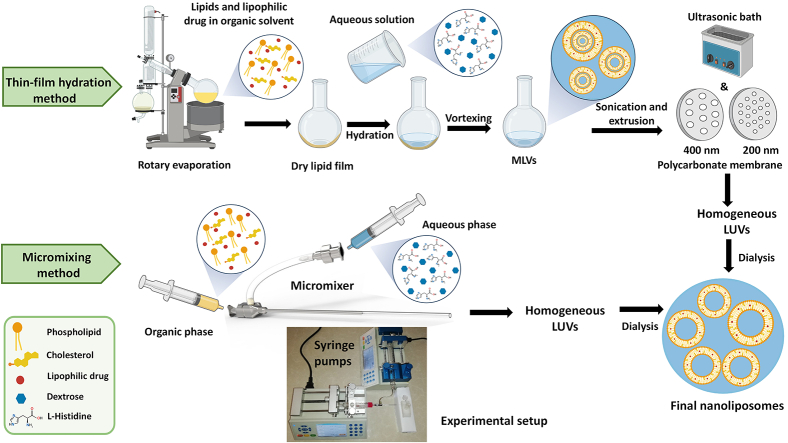

## 1 Introduction

The use of successful nanoliposomal formulations in clinics is sometimes limited due to poor translation of conventional laboratory techniques to mass production procedures. The well-known and acceptable method for manufacturing of nanoliposomes is thin-film hydration/extrusion (TFH) method, which needs to be re-optimized for clinical applications (Shah et al., 2019; Shepherd et al., 2021; Valencia et al., 2012). Conventional production of nanoliposomes is generally based on the self-assembly of lipids in the bulk phase, which often requires post-processing steps such as sonication, extrusion, freeze-thaw or high-pressure homogenization. Thus, these methods often result in batch-to-batch variations and poor product quality control (Balbino et al., 2017; Ran et al., 2016; Yu et al., 2009). Compared to bulk methods, continuous synthesis of liposomal nanocarriers (NCs) usually provides higher levels of controllability and reproducibility for modulating the physicochemical properties of NCs (Aghaei et al., 2021; Ekonomou et al., 2022; Matsuura-Sawada et al., 2023). In this way, the production of nanoliposomes using microfluidic approach presents a feasible and effective method that can provide a reliable technology to fine control the mixing of formulation solutions (Xiu et al., 2024). Microfluidics allows for the rapid mixing of two or more liquid streams, e.g. an organic phase that is a mixture of lipids in a solvent and an aqueous phase, in a confined space of a microchannel in a continuous manner (Hibino et al., 2019; Nguyen et al., 2019; Webb et al., 2020). In contrast to top-down methods for nanoliposome manufacture, microfluidic devices based on the nanoprecipitation approach can produce nanoliposomal carriers in a one-step process (Joshi et al., 2016; Liu et al., 2022; Niculescu et al., 2021). Microfluidics allows for producing NCs using low volumes of precursors; via precise control of their flow rates that creates high batch-to-batch reproducibility (Forbes et al., 2019; Haghighi et al., 2024; Weaver et al., 2024). In addition, microfluidics enables high-throughput liposome production owing to its easy scale up that is rapidly adopted by the pharmaceutical industry to reduce production costs and time (Li and Jiang, 2018; Lim et al., 2020; Maeki et al., 2018).

Various configurations of micromixers including staggered herringbone (Cheung and Al-Jamal, 2019; Vigario et al., 2022), bifurcating (Webb et al., 2020), serpentine and Y-shaped (Bottaro et al., 2017; Ghodke et al., 2023), baffles (Kimura et al., 2018) and hydrodynamic flow focusing have been explored for production of liposomes. In this way, microfluidic hydrodynamic flow focusing (HFF) in 2D and 3D configurations (Hood et al., 2014; Jahn et al. (2015); Ran et al., 2016), provides high flexibility for liposome production. However, the majority of these microfluidic mixers are made from polydimethylsiloxane (PDMS) or some thermoplastics that have limited solvent compatibility (Alavi et al., 2024; Costa et al., 2021; Jaradat et al., 2021). While, the hydrophobic nature of certain phospholipids, such as phosphatidylserine (PS) and phosphatidylethanolamine (PE), makes them poorly soluble in less corrosive solvents, necessitating the use of more aggressive organic solvents, like tetrahydrofuran (THF) (Dervichian, 1964). The fabrication of microfluidic devices resistant to such solvents is often complex and requires specialized equipment. Therefore, some researchers have used off-the-shelf materials to create simpler, yet functional micromixers for synthesizing liposomes (Lim et al., 2014; Vladisavljević et al., 2014). However, existing studies using such micromixers for liposome synthesis face several limitations including difficult assembly, lack of drug encapsulation studies, absence of in vitro cell-based tests, e.g., cytotoxicity and cellular uptake, and no comparative analysis with traditional techniques like thin-film hydration method. For instance, one study achieves high-throughput liposome synthesis but only encapsulates dyes instead of therapeutic drugs, and lacks in-depth in vitro tests and comparative analyses (Lim et al., 2014). Another study introduced a coaxial micromixer using an off-the-shelf device; however, the design was not reproducible due to the fabrication method and modifications applied to the off-the-shelf components. Furthermore, while they compared the physicochemical properties of the liposomes with those produced by the traditional ethanol injection method, their study lacked drug encapsulation and in vitro tests on the liposomes (Vladisavljević et al., 2014).

**Fig. 1:**
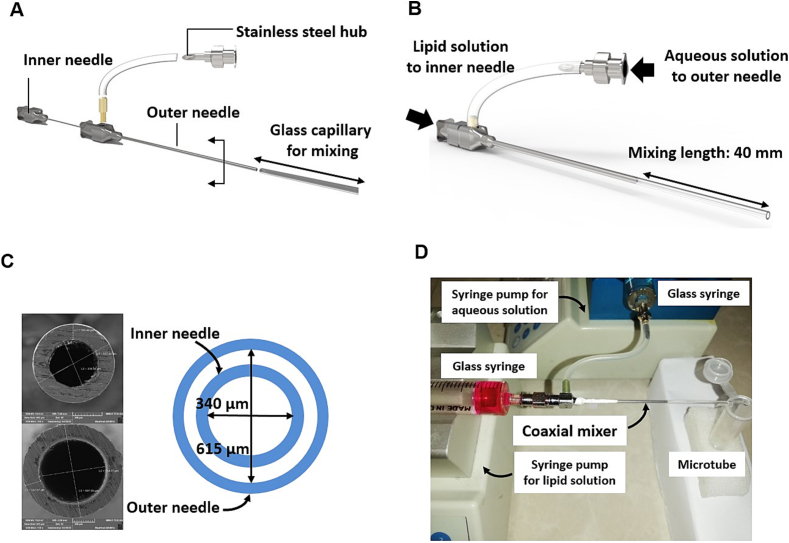
Coaxial micromixer. (A) Schematic illustration for the components of the coaxial micromixer, (B) schematic of the assembled micromixer, (C) SEM image of the needle cross-section to show their diameters, (D) image of the experimental setup.

To address these gaps, this research introduces a coaxial micromixer made from off-the-shelf materials that are compatible with a wide range of solvents, including highly corrosive ones like tetrahydrofuran (THF). By leveraging the high compatibility of steel combined with glass, this device is designed to withstand harsh chemical environments. In this micromixer, similar to HFF micromixer (Jaradat et al., 2023; Riahi et al., 2015), one stream flows at the center of the microchannel that is surrounded by another stream, allowing rapid mixing of lipid solution with buffer phase in a HFF configuration. The micromixer was fabricated through coaxial assembly of two low-cost off-the-shelf metallic needles, Fig. 1(A-B). Lipid solution, prepared in THF, was introduced to the mixer via the inner needle while the aqueous phase was pumped to the mixer through the outer needle to surround the lipid stream, Fig. 1(C-D). This device could be used many times benefiting from easy disassembly, cleaning and reassembly. The device benefits from coaxial mixing of precursors. Self-assemblies of lipids to form nanoliposomes occur at the interface of lipid and aqueous phases that could be controlled through adjusting the flow rates of the streams and their flow rate ratio.

In this research, atorvastatin calcium (ATC) was used for liposomal encapsulation. ATC as a lipophilic statin is used to treat hypercholesterolemia. In addition, its anticancer, antioxidant and anti-inflammatory activity has been shown (Kumar et al., 2016). However, statins have limitations such as low bioavailability, non-specific delivery and side effects. Various strategies including the use of nanoparticles have been developed to improve the bioavailability, enhance the delivery efficacy and thus reduce the side effects of statins (Akl et al., 2023; Li et al., 2019). Many studies have investigated different nanoparticles such as micelles (Xu et al., 2014), SLNs (Gaber et al., 2024), polymers (Grune et al., 2021), and liposomes (Thomas et al., 2024) (Akl et al., 2024) to increase the bioavailability, targeted delivery and enhance the therapeutic effects of ATC. Although there are many researches in this field, the liposomal form of ATC has not been synthesized by microfluidic method. We produced nanoliposomes containing ATC by a one-step and cost-effective micromixing approach and compared them with the thin film hydration method.

Firstly, numerical simulations of mixing process between two solutions of the formulation were conducted to understand the mixing behavior. This was followed by the implementation of a design of experiments (DOE) approach based on Response Surface Methodology (RSM) to investigate the effect of various input parameters including total flow rate (TFR), flow rate ratio (FRR), and lipid concentration on liposome characteristics, i.e. size, PDI, and zeta potential (Khorshid et al., 2023). Moreover, this study provides a comprehensive analysis by comparing the proposed micromixer with the established thin-film hydration method, Fig. S1. To examine the capacity of the coaxial micromixer in the production of nanocarriers, empty and ATC-loaded liposomes were prepared using TFH and micromixing methods and then compared in terms of size, PDI, zeta potential, encapsulation efficiency (*EE*%), and dug release profile. In addition, the stability, cellular uptake, and cytocompatibility of nanoliposomes produced using both methods were evaluated.

## 2 Materials and methods

### 2.1 Fabrication process

To fabricate the coaxial micromixer, two off-the-shelf stainless steel hub blunt point biopsy needles (Luer Lock) in different sizes (20 G and 23 G) were used. The smaller needle with 340 μm and 557 μm inner diameter (I.D.) and outer diameter (O.D.), respectively, was inserted through the larger one (615 μm I.D. and 897 μm O.D.), with the same length (45 mm), Fig. 1. The larger needle was modified by drilling a hole in the top of the needle hub and threaded by a tap. A brass connector was inserted through the hole and connected the large needle to the silicone tube. The silicone tube was then attached to another stainless steel hub. The outer needle, i.e. the larger one, was connected to a glass capillary and sealed with Teflon thread seal tape. The wetted materials of this micromixer include stainless steel, glass, silicone, and brass, which are compatible with the employed solutions. Fig. 1(A-B) illustrates the exploded view of the fabricated coaxial micromixer and the assembled micromixer with dimensional details, respectively. The dimensions of the cross-section for both needles were measured by scanning electron microscopy (SEM) images, Fig. 1(C).

The micromixing setup presented in our study is cost-effective. The main components include two stainless steel needles, which can be purchased from AliExpress for around $8 each, totaling approximately $16. Additionally, the needle hub and silicone tubing cost $5 combined. The labor cost for machining a connector for the inner needle and creating a hole in the outer needle to accommodate a connector, and cutting additional length of the needles should be around $100. The whole device could be assembled easily requiring no special expertise in less than 15 min. In addition, two syringes along with one or two syringe pumps are required for the injection of solutions that are easily accessible in any microfluidic lab. Thus, this cost-efficient setup highlights the accessibility and practicality of our design for various experimental applications.

### 2.2 Numerical study

Numerical modeling was performed to get insight into the mixing performance of the coaxial micromixer. The following is a complete description of the numerical simulation, including model geometry, governing equations, and numerical solution method.

#### 2.2.1 Micromixers geometry and governing equations

**Fig. 2:**
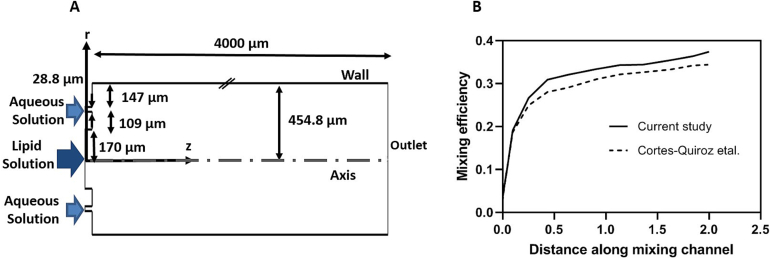
(A) Schematic illustration of the overall computational domain; cylindrical symmetry was applied to the computational domain, (B) the comparison of mixing efficiency of T-shape micromixer (Aspect ratio = 0.5) in *Re* = 250 with the results of Cortes-Quiroz study.

Based on the maximum employed flow rate, i.e. 1500 μL/min, the Reynolds number (the indication of flow regime, calculated with *Re* = $\frac{\mathrm{\rho U}D_{h}}{\mu }$, where ρ, U, $D_{h}$ and μ are density, inlet velocity, the hydrodynamic diameter, and viscosity, respectively) was less than 100, thus the fluid flow was considered laminar (Gamrat et al., 2008). The micromixer geometry was cylindrical, and thus, the governing equations in the cylindrical coordinate system (r, θ, z) were solved. Due to the axisymmetric nature of the geometry around the central axis, the governing equations mentioned in supplementary information were independent of θ, simplifying the model from 3D to 2D. This reduction in complexity facilitated the numerical simulation and enhanced the understanding of fluid behavior within the micromixer. Fig. 2(A) shows the schematic of the computational domain and the dimensions as well as the coordinate system (r, z). Lipid composition dissolved in THF entered the micromixer from the central needle with an inner radius of 170 μm, and an aqueous solution from the outer needle with an inner and outer radius of 279 μm and 307 μm, respectively. Both solutions finally entered a glass capillary with an inner radius of 454 μm and a mixing length of 4000 μm.

It was assumed that the fluid flow through the mixer had steady-state and two-dimensional (2D) conditions, and behaved as a Newtonian fluid with constant properties. For detailed information on the governing equations and boundary conditions utilized in the numerical simulations, please refer to the Supplementary Information section. This section provides comprehensive insights into the mathematical models governing the mixing behavior within the coaxial micromixer. Additionally, it outlines the conditions at the inlets and outlets of the micromixer, along with boundary conditions applied to the micromixer walls and axis. Moreover, the calculation of mixing efficiency is elucidated, offering a deeper understanding of the evaluation metrics employed in the study.

#### 2.2.2 Numerical solution procedure

Using conservation equations and appropriate boundary conditions presented in supplementary information, a numerical model was developed to analyze the fluid flows and mixing characteristics through the coaxial micromixer. It is important to note that the second-order upwind strategy and SIMPLEC (Jang et al., 1986) were used for discretizing the variable's gradient in governing equations and pressure-velocity coupling, respectively.

The impact of four mesh numbers including 182,000, 365,000, 735,000, and 1,420,000, on the mixing performance was assessed to study the grid-independency. In the findings of Table S1 in the supplementary information, the third mesh number was chosen to conduct the simulations since the impact of mesh numbers on the mixer's overall performance is minimal in mesh numbers beyond 735,000.

To assess the accuracy of the developed model, the mixing efficiency of the T-shaped micromixer along the micromixer path at Reynold number of 250 was compared with the results of Cortes-Quiroz et al. (Cortes-Quiroz et al., 2014) and depicted in Fig. 2(B). The acquired results are very consistent with their findings, demonstrating the numerical model's high degree of precision.

### 2.3 Experimental study

#### 2.3.1 Materials

To compare the performance of the coaxial micromixer with the TFH method, liposomes were produced using both methods. Lipids 1, 2-dioleoyl-sn-glycero-3-phosphatidylcholine (DOPC), 1, 2-dioleoyl-sn-glycero-3-phosphatidylserine (DOPS) and cholesterol (Chol) were purchased from Avanti polar lipids Inc., USA (purity >99 %). Methanol, chloroform, and tetrahydrofuran (THF), all HPLC grade, were purchased from Merck, Germany. Atorvastatin calcium (ATC) was purchased from Sami Saz Pharmaceutical Company, Iran. Histidine was purchased from Merck, Germany. Dialysis tubing cellulose was purchased from Sigma Aldrich Company Ltd., Poole, UK. Fluorescent dye of 1,1′-dioctadecyl-3,3,3′,3′-tetramethylindotricarbocyanine iodide (DiR) was purchased from Invitrogen, Carlsbad, CA. DMEM culture media and fetal bovine serum (FBS) were purchased from Gibco (Carlsbad, CA). 3-(4,5-dimethylthiazol-2-yl)-2,5-diphenyl tetrazolium bromide (MTT) was obtained from Sigma-Aldrich (Taufirchen, Germany).

#### 2.3.2 Preparation of liposomal formulations using thin-film hydration/extrusion method

Nanoliposomes containing DOPC, DOPS, and cholesterol in the optimized ratio (35:25:40 respectively) were prepared using thin-film hydration/extrusion technique (Mansury et al., 2019; Yazdi et al., 2020; Zhang, 2017). Lipids were dissolved in chloroform with a final concentration of 48 mM. To prepare drug-containing liposomes, ATC with the concentration of 5 % dissolved in methanol was added to the mixture of cholesterol and phospholipids (DOPC:DOPS:Chol:ATC) with a molar ratio of 30:25:40:5 respectively. The solvent was removed using a vacuum rotary evaporator and the resulting lipid thin-film was freeze-dried for 2 h. The obtained lipid thin-film was hydrated at 37 °C with histidine/dextrose buffer (10 mM, pH 6.5) and then vortexed, sonicated, and extruded via polycarbonate membrane (400 and 200 nm) to reduce the size and lamellarity of the NCs. Free ATC was removed through dialysis in histidine/dextrose buffer.

#### 2.3.3 Preparation of liposomal formulations using the coaxial micromixing method

In this study, due to the insolubility of DOPS in solvents such as ethanol, methanol, propanol, or butanol, we used THF which is a water-miscible solvent as a lipid phase solvent. To prepare liposomes with a total lipid concentration of 48 mM, the organic phase containing DOPC, DOPS and cholesterol (with a molar ratio of 35:25:40, respectively) and also for drug-encapsulated liposome, ATC with the concentration of 5 % dissolved in THF. The lipid blend in THF solution and the aqueous phase containing histidine/dextrose buffer (10 mM, pH 6.5) were used as inner and outer streams, respectively. To introduce streams to coaxial micromixer with controlled flow rates, two syringe pumps were used (Chemyx Fusion 200 Syringe Pump). The micromixer has a coaxial cylindrical geometry in which lipid mix precursors, i.e. organic phase, and aqueous phase are injected through the inner and outer needle using two glass syringes, respectively, Fig. 1(D). Suitable total flow rate (TFR) and flow rate ratio (FRR) for pumping the solutions were obtained from the numerical simulation and DOE approach. The output stream from the coaxial mixer was collected. Dialysis was then performed to remove excess THF and unloaded drug.

#### 2.3.4 Design of experiments (DOE)

To efficiently explore the relationships between multiple input factors and their impact on liposomes properties, a Design of Experiments (DOE) approach using Box-Behnken Design (BBD) was employed. This approach minimizes the number of required tests in Response Surface Methadology (RSM) while maximizing the information obtained, thereby reducing both time and cost.

Design Expert software Version 13 (Stat-Ease, Inc., USA) was utilized for this purpose. The BBD is considered the most favorable method for developing predictive models involving three or more input variables (Guerreiro et al., 2021). In this way, BBD was used to assess the effects of three independent variables including FRR, TFR, and lipid concentration (LC), on size, PDI, and zeta potential of nanoparticles (Rebollo et al., 2022; Sedighi et al., 2019). For each independent variable, low, medium, and high levels were defined as shown in Table 1. The ranges for TFR and FRR were based on insights obtained from the numerical study to validate and analyze their impact on liposome size. The FRR was limited to 1:3, as ratios beyond 1:4 were found to reduce liposome yield. The lipid concentration range was chosen based on the maximum concentration used in thin-film hydration method, with reductions to two-third and one-half to assess its effect on liposome physicochemical characteristics. A total of 17 experiments were planned, and each run was made in triplicate, and nanoparticle size, PDI and zeta potential were measured. A polynomial regression model was conducted to establish the relationship between the selected independent and dependent variables.

**Table 1:** The independent variables in DOE.

Independent variables	Units	Levels		
FRR (A)	–	1	2	3
TFR (B)	μL/min	150	825	1500
Lipid concentration (C)	mM	24	36	48

#### 2.3.5 Liposome characterization

The dynamic light scattering (DLS) was used to determine the size and the polydispersity index (PDI) of the liposomes produced by both methods (Malvern Zetasizer Nano-ZS, Malvern Instruments, Worcs., UK). The measurement of vesicle size and PDI were performed at 25 °C in histidine/dextrose buffer. Liposome surface charge was measured in 10 mM NaCl solution using the Malvern Zetasizer Nano-ZS. All measurements were performed in triplicates.

#### 2.3.6 Transmission electron microscopy (TEM)

To prepare the liposomal sample for morphological imaging, 10 μL of the liposomal samples prepared using both techniques were diluted 1:100 with hydration buffer and then a drop of the diluted sample was placed on a carbon-coated copper grid. The sample was given time to dry completely. Next, negative staining was done on the thin layer of the sample. In this way, a quantity of uranyl acetate dye 2 % (*W*/*V*) with pH 4.5 was added to the sample placed on the grid. Then the final stained sample was exposed to the ambient air to dry completely. Finally, nanoliposomes were visualized by a transmission electron microscope (Zeiss, Leo 912AB, Germany) with a voltage of 120 kV (63).

#### 2.3.7 Determination of ATC encapsulation in nanoliposomes

The amount of ATC encapsulated in nanoliposomes manufactured by TFH and the micromixing methods was determined by UV spectrophotometer. For this purpose, ATC-containing liposomes were lysed in methanol and the absorbance of ATC at the maximum wavelength (λ max = 246 nm) was read by a spectrophotometer. To eliminate the effect of lipid absorption, the empty liposomes lysed in methanol were considered as blank. ATC concentration was determined by the drug calibration curve, i.e. absorbance against different concentrations of ATC. Finally, the encapsulation efficiency percentage (*EE%*) was calculated using Eq. (1) (Arduino et al., 2024; Hamedinasab et al., 2020):

(1) $$\mathrm{EE}\%=\frac{\mathrm{ATC}\;\mathrm{concentration into the liposomes after dialysis}\;}{\;\mathrm{ATC}\;\mathrm{concentration initially added}}\times 100\;$$

#### 2.3.8 Phosphate assay

To determine the exact amount of phospholipids in liposomes produced by both methods, phosphate assay was performed. Bartlett's method is based on determining the amount of phosphate in the sample by colorimetric method (Bartlett, 1959; Torchilin et al., 2003). The phosphate content of liposomes can be traced after the degradation of phospholipids with oxygenated water and its conversion to mineral phosphate. Amino 2-naphthyl 4-sulfonic acid is absorbed at a wavelength of 800 nm (Farzaneh et al., 2018).

#### 2.3.9 Differential scanning calorimetry (DSC)

The phase transition temperature (T_m_) of liposomal formulations was measured using a DSC823e calorimeter (Mettler Toledo, Switzerland). Six single-use aluminum pans with caps and one empty pan, as reference, were used. Empty and ATC-containing liposomes with a total concentration of 48 mM prepared by TFH and the micromixing methods were used for DSC measurements. Data were collected in the temperature range of −20 to 60 °C and with a thermal scan speed of 1 °C/min. STARe V9.00 software from Mettler Toledo was used to convert raw data to molar heat capacity (MHC). About 25 mg per sample was used for DSC measurement (Alavizadeh et al., 2017).

#### 2.3.10 Stability study of empty liposomes

The stability of empty liposomes made by both TFH and needle micromixer techniques was studied for one month at 4 °C, 25 °C and 37 °C. Three different batches of empty liposomes were prepared by both methods. On days 1, 8, 15, 22, and 29, some samples were taken and evaluated in terms of size, PDI, and surface charge, as well as the appearance characteristics of nanoparticles.

#### 2.3.11 In vitro release study

The in vitro release profile of ATC from nanoliposomal formulations was analyzed by dialysis technique. One mL of the drug-containing liposomes produced by both methods were placed in a dialysis bag (cut off 12 kDa) and were immersed in 100 mL of histidine/dextrose buffer (pH 6.5) at 37 °C with mild stirring. Sampling from the dissolution medium was carried out at various time points of 0.5, 1, 2, 4, 6, 8, 10, 24, and 48 h and ATC quantified by UV spectrophotometer (Jayasundara et al., 2021). 1 mL of the withdrawn dissolution medium was replaced with 1 mL of fresh histidine/dextrose buffer. The cumulative release was calculated using the following Eq. (2):

(2) $$\mathrm{Mt}\left(n\right)=\mathrm{Vr}.\mathrm{Cn}+\mathrm{Vs}.Ʃ\mathrm{Cm}\;$$

where $\mathrm{Mt}\left(n\right)$ corresponds to the current cumulative mass of released ATC at time t, n is the number of samplings, Cn is the current concentration of ATC in the medium, ƩCm is the summed total of the previously measured concentrations, Vr is the release medium volume, and Vs refers to the sample volume removed to conduct the assay (Mirhadi et al., 2022; Solomon et al., 2017).

#### 2.3.12 Cell viability

J774 macrophage cell line was cultured in DMEM medium containing 10 % FCS and 1 % antibiotic (penicillin and streptomycin) at 37 °C and 5 % CO_2_. The cytocompatibility assessment of empty and drug-containing liposomal formulations prepared by both TFH and micromixing methods was performed using the MTT cytotoxicity test. Into a 96-well plate, 5000 cells were seeded per well. Following 24 h of incubation at 37 °C and 5 % CO_2,_ the cells were treated with various concentrations of empty and ATC-loaded liposomes prepared by both methods. After 48 h of incubation, the medium of each well was replaced with 10 μL of MTT in 100 μL of culture medium and incubated for 4 h. Then the medium containing MTT was discarded and 200 μL of DMSO was added to each well and placed on the shaker for 20 min. Finally, the absorption of the samples was measured at 570 nm by a microplate reader (Awareness Technology Inc., USA). The percentage of cell viability was calculated by the following Eq. (3).

(3) $$\mathrm{Cell viability}\%=\frac{A\;\mathrm{treatment}-A\;\mathrm{blank}\;}{\;A\;\mathrm{control}-A\;\mathrm{blank}}\times 100\;$$

where *A treatment* and *A control* refer to the absorbance of the treated and untreated cells, respectively. *A blank* is the absorbance of the culture medium without cells.

#### 2.3.13 Uptake of nanoliposomes by the J774 macrophage cell line

Empty and ATC-loaded liposomes were prepared by TFH and the micromixing methods. DiR fluorescent dye was added to the lipid composition of each of the liposomes with a molar ratio of 0.2 %. To investigate the uptake of empty and ATC-containing nanoliposomes by the J774 macrophage cell line, 5 ✕ 10^5^ cells per well of 12-well plate were seeded with DMEM culture medium. After 24 h of incubation at 37 °C and 5 % CO_2_, macrophage cells were treated with nanoliposomes for 4 h. Also, untreated cells were used as control. Next, the cells were separated from the bottom of the wells and transferred to flow cytometry tubes. Then they were washed twice with staining buffer (*PBS* with *2 % FCS*) and centrifuged at 1600 RPM for 5–6 min. Finally, 500 μL of staining buffer was added to each tube and read with a flow cytometry device, and the results were analyzed with FlowJO software version 10.5.3.

#### 2.3.14 Statistical analysis

All experiments were performed in triplicates with the calculation of means and standard deviations (Mean ± SD). Statistical analyses were accomplished by the 8.4.3 version of GraphPad Prism software (San Diego, CA). Unpaired *t*-test and one-way ANOVA test were used for determination of statistical significance. *P* value ≤0.05 was considered to be significant.

## 3 Results and discussion

### 3.1 Numerical results

Microfluidics has recently opened a new procedure for manufacturing nanoliposomes through reproducible mixing of materials in milliseconds at the nanoliter scale (Al-Amin et al., 2020; Kastner et al., 2014). The hydrodynamic conditions impact the mixing efficiency through the coaxial micromixer with critical effects on particle formation. To this end, firstly, the numerical results were provided to determine the effect of TFR, i.e. the summation of the flow rates of the aqueous and lipid solutions, and the FRR, i.e. the ratio of the aqueous solution flow rate to lipid solution flow rate, on mixing efficacy. Then, the experimental results of the liposomes' synthesis in the coaxial micromixer at a certain TFR and FRR were discussed.

**Fig. 3:**
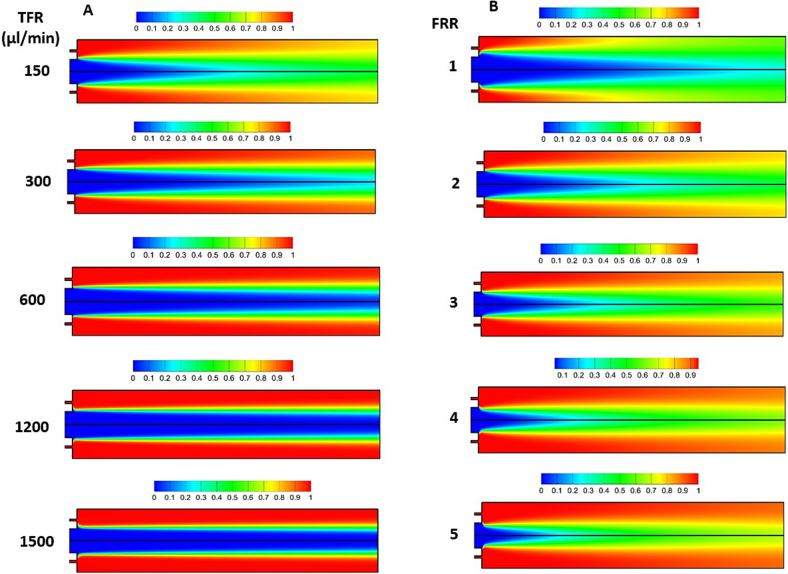
Numerical simulation of the mixer. (A) effect of TFR on the normalized concentration distribution of aqueous solution through the coaxial micromixer at FRR of 2, (B) effect of FRR on the normalized concentration distribution of aqueous solution through the coaxial micromixer at TFR = 150 μL/min. To conduct the numerical simulations, the concentration of the inlet solution for the inner needle (central needle) was set at zero, while the concentration of the inlet solution for the outer needle was set at one.

Fig. 3(A) demonstrates the distribution of normalized concentration inside coaxial micromixer at FRR = 2 and TFRs of 150 μL/min to 1500 μL/min. The findings show that as TFR increased, the mixing performance rapidly dropped; while at TFRs greater than 600 μL/min, the mixing was only limited to the interface of the solutions. This is because of diffusion dominant nature of mixing in the presented micromixer. In this way, a high residence time through the mixing channel, resulting from the low velocity of the two streams, produces a higher mixing index. However, the diffusion effect on mixing decreased gradually as TFR increased, resulting in lower mixing. Similar outcomes were noted in the T-shaped micromixer, where diffusion plays a dominant role (Akar et al., 2021; Chen et al., 2017). Thereby, a TFR of 150 μL/min was considered suitable to perform the other simulations.

The next effective parameter is FRR. Fig. 3(B) depicts the effect of several FRRs on the normalized concentration distribution of aqueous solution inside the coaxial micromixer at TFR = 150 μL/min. It is observed that as FRR increases, the region around the center of the mixing channel experiences higher and faster mixing, while the regions near the wall have less mixing, especially at the high FRR of 5. Owing to the fact that fast and uniform mixing is desired to produce small size liposomes with low PDIs; FRR of 1 and 2 could have optimum performance. At these FRRs, relatively fast mixing was observed, not only around the core but also near the wall of the micromixer. Thus, based on numerical simulations, TFR of 150 μL/min with FRR of 1 and 2 show more uniform and fast mixing and probably may result in production of more homogenous liposomes with appropriate size. To gain a deeper understanding of the TFR and FRR, alongside lipid concentration—whose impact cannot be directly observed through mixing simulations—a DOE approach along with RSM was conducted in the subsequent section. This approach aimed to validate the relationship between the mixing of the two solutions and the characterization of liposomes, including size, PDI, and zeta potential.

### 3.2 Experimental results

#### 3.2.1 DOE study to investigate the effects of micromixing inputs on liposomecharacteristics

##### 3.2.1.1 Analysis

The results of particle size, PDI, and zeta potential of 17 experimental runs are illustrated in Table 2. The reduced cubic model was applied to the particle size response with a square root transformation, while a quadratic model with an inverse transformation was used for PDI. For zeta potential, a quadratic model was employed without any transformation. These models were chosen as they provided the best fit for the data, as determined through the RSM analysis. The deployment of these models ensures that the relationships between the input parameters and the responses are accurately captured, enabling a comprehensive understanding of the effects of the independent variables on liposome characteristics. The full ANOVA tables for the three outputs along with the predicted equations are shown in Table S2 in the supplementary information file. The results of the ANOVA analysis show that the *p*-value of the model is <0.05 for all three responses investigated. Additionally, the predicted R^2^ and adjusted R^2^ are in reasonable agreement, with a difference of less than 0.2, indicating that the regression model can predict the responses effectively without overfitting. Adequate precision values were also satisfactory for all three characteristics, exceeding the threshold of 4 (Saberi et al., 2024). Furthermore, the plots of actual versus predicted values for each response (Fig. S2 in the supplementary information) demonstrate good agreement, confirming that the proposed model is suitable for data analysis and optimization of parameters.

**Table 2:** The result of DOE design with Box-Behnken design pattern, demonstrated as mean ± S. D. (*n* = 3).

Run	FRR	TFR (μL/min)	LC (mM)	Size (nm)	PDI	Zeta potential (mV)
1	2	825	36	343 ± 5.93	0.23 ± 0.03	−56.53 ± 1.55
2	2	1500	24	225.17 ± 19.44	0.41 ± 0.04	−53.8 ± 9.05
3	3	1500	36	84.14 ± 8.52	0.22 ± 0.02	−36.47 ± 4.69
4	1	150	36	143.2 ± 6.93	0.1 ± 0.0	−28.9 ± 4.41
5	1	825	24	117.23 ± 8.36	0.15 ± 0.06	−37.3 ± 3.28
6	3	150	36	136.93 ± 11.79	0.29 ± 0.04	−24.93 ± 7.95
7	1	825	48	137.4 ± 8.77	0.12 ± 0.01	−41.5 ± 3.54
8	2	150	24	198.63 ± 13.9	0.25 ± 0.04	−42.7 ± 6.47
9	1	1500	36	155.1 ± 5.8	0.13 ± 0.0	−33.13 ± 1.4
10	2	150	48	187.23 ± 7.28	0.16 ± 0.03	−56.67 ± 2.87
11	2	825	36	341.86 ± 6.4	0.21 ± 0.01	−55.7 ± 3.37
12	2	825	36	349.24 ± 9.6	0.22 ± 0.03	−56.92 ± 4.55
13	3	825	48	112.43 ± 7.99	0.25 ± 0.0	−62.3 ± 7.08
14	3	825	24	58.76 ± 9.82	0.27 ± 0.05	−29.5 ± 4.57
15	2	1500	48	526.0 ± 24.04	0.3 ± 0.0	−69.33 ± 3.3
16	2	825	36	354.65 ± 5.21	0.24 ± 0.02	−57.55 ± 5
17	2	825	36	359.1 ± 9.05	0.24 ± 0.02	−58.43 ± 1.9

##### 3.2.1.2 Optimization

In light of investigating the immunotolerogenic effects of these nanoparticles and their targeting to antigen-presenting cells (APCs) in future studies, a size of between 150 and 200 nm and a high negative charge, around −50 to −60 mV, were considered optimal (Dangkoub et al., 2021; Li et al., 2024; Lin et al., 2023; Thorp et al., 2020). Additionally, PDI values below 0.2 indicate the homogeneity of the nanoliposomes (Danaei et al., 2018). By applying constraints, i.e. goals to the dependent and independent variables, the optimal liposome formulation was developed.

**Fig. 4:**
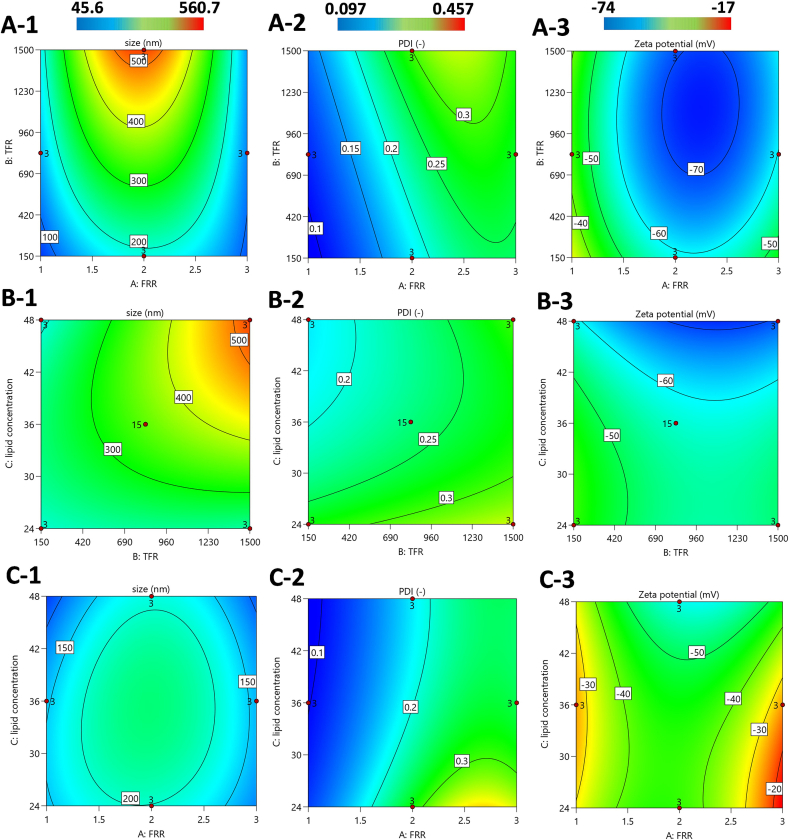
Impact of input parameters of TFR, FRR, and lipid concentration on size, PDI, and zeta potential of the liposomes: A) TFR and FRR at LC = 48 mM, B) LC and TFR at FRR = 2, C) LC and FRR at TFR = 150 μL/min.

Fig. 4 illustrates the impact of two independent variables, A: TFR and FRR at lipid concentration (LC) = 48 mM, B: LC and TFR at FRR = 2, C: LC and FRR at TFR = 150 μL/min on the size, PDI, and zeta potential of the liposomes. As shown in Fig. 4(A-1), it is evident that increasing the TFR results in a significant increase in both the size and PDI of the liposomes. For example, as the TFR rises from 150 μL/min to 1500 μL/min, the liposome size increases substantially, from 170 nm to over 525 nm. This trend is in agreement with our numerical simulations, which revealed that at higher flow rates, the two solutions flow through the micromixer at a faster speed, leading to reduced interaction for mixing. Consequently, poor mixing occurs, as its effect is reflected in the formation of larger and less uniform liposomes.

Examining the effect of FRR on liposome characteristics, it can be observed that at lower FRR values i.e. 1 and higher FRR i.e. 3, smaller liposomes are produced, around 100 nm. However, while both FRR 1 and 3 result in the production of smaller liposomes, FRR 3 leads to a higher PDI shown in Fig. 4(A-2), indicating less uniformity. On the other hand, an FRR of 2 yields liposomes with more desirable size and PDI, and highly negative zeta potential. Furthermore, these findings are consistent with the numerical simulations, which show that FRR values of 1 and 2 create more homogeneous mixing, whereas an FRR of 3 leads to poorer mixing, contributing to the formation of less uniform liposomes.

As for the zeta potential contour illustrated in Fig. 4(A-3), the blue regions represent more favorable zeta potentials. Since zeta potential reflects the electrostatic repulsion between particles, more negative zeta potential values are more favorite for long-term stability of liposomes, as they repel each other more strongly, preventing aggregation. Zeta potential values around −50 mV are ideal, as they indicate superior stability. Therefore, a combination of an FRR of 2 and a TFR of 150 μL/min leads to highly stable liposomes with a zeta potential closer to −50 mV.

When considering the interaction between lipid concentration with TFR, and FRR, reflected in Fig. 4B and C respectively, similar trends emerge. As shown in Fig. 4(B-1), higher lipid concentrations correlate with larger liposome sizes, particularly at higher TFR values. This result aligns with findings in the literature (Akar et al., 2024), such that as the lipid concentration increases, more lipid molecules are present, leading to the formation of larger liposomes. Although higher TFRs lead to a significant increase in liposome size with higher lipid concentrations, at lower TFRs (e.g., 150 μL/min), the effect of lipid concentration on liposome size is minimal. The impact of lipid concentration on zeta potential is also notable. As the lipid concentration increases, the zeta potential becomes more negative due to higher number of negatively charged phospholipids. This is one of the reasons why selecting a higher lipid concentration is favorable, as it contributes to improved electrostatic stability and higher liposome yield. Fig. 4C illustrates the effect of lipid concentration and FRR at a TFR of 150 μL/min, showing a similar relationship between the inputs as described in detail above.

##### 3.2.1.3 Validation

The best parameters for the production of nanoliposomes were identified as FRR of 2, TFR of 150 μL/min, and lipid concentration of 48 mM. To validate the design of experiments (DOE), experimental measurements of size, PDI, and zeta potential obtained with these selected parameters were compared to the predicted values (Table 3). The close agreement between actual and predicted values confirms the effectiveness of the Box-Behnken design in predicting the optimal conditions for nanoparticle production (Tatode et al., 2018).

**Table 3:** Comparison of predicted and observed values for size, PDI and zeta potential.

Response	Predicted value	Observed value
Size (nm)	187.66 ± 19.32	187.23 ± 7.28
PDI	0.181042 ± 0.02	0.16 ± 0.03
Zeta potential (mV)	−58.6042 ± 5.83	−56.67 ± 2.87

#### 3.2.2 Physicochemical properties of nanoliposomes: A comparative analysis between TFH and micromixing methods

In this comparative study, liposomes were generated by the coaxial micromixer and conventional thin-film hydration followed by extrusion method. The light scattering (DLS) analysis showed that both methods could yield unilamellar vesicles with similar size, PDI, and surface charge (Table 4). As illustrated in Table 4, the average sizes of empty and drug-loaded liposomal formulations prepared using both approaches are within the range of 180–190 nm and 150–170 nm, respectively. A non-significant increase in the size of liposomes produced by the micromixer was observed compared to TFH method (*P* > 0.05). The presence of ATC in liposomes made by TFH and micromixing methods has reduced the size of these nanoparticles due to the hydrophobic interactions between the drug and phospholipids, which leads to the compaction of nanoliposomes (Naeini et al., 2021).

**Table 4:** Size, polydispersity index (PDI) and zeta potential of liposomal formulations either empty (L(Empty)) or drug-loaded (L(ATC)). Also, encapsulation efficiency (*EE%*) and final concentration of ATC (C) prepared by TFH (F) and micromixing ((M), TFR = 150 μL/min and FRR = 2) methods at 25 °C, demonstrated as mean ± S. D. (n = 3).

Method	Liposomal formulation	Size (nm)	PDI	Zeta potential (mV)	*EE%*	C (mg/mL)
TFH	L(Empty)_F_ (DOPC:DOPS:CHol) (35:25:40)	181 ± 2.8	0.085 ± 0.006	−50.55 ± 4.1	–	–
	L(*ATC*)_F_ (DOPC:DOPS:CHol:ATC) (30:25:40:5)	157.02 ± 5	0.163 ± 0.02	−49.3 ± 4	67.7 ± 0.00	1.85
Micromixing	L(Empty)_M_ (DOPC:DOPS:CHol) (35:25:40)	189.2 ± 10.2	0.159 ± 0.016	−57 ± 5.6	–	–
	L(*ATC*)_M_ (DOPC:DOPS:CHol:ATC) (30:25:40:5)	167.8 ± 7	0.173 ± 0.016	−63 ± 0.25	70 ± 0.04	1.93

**Fig. 5:**
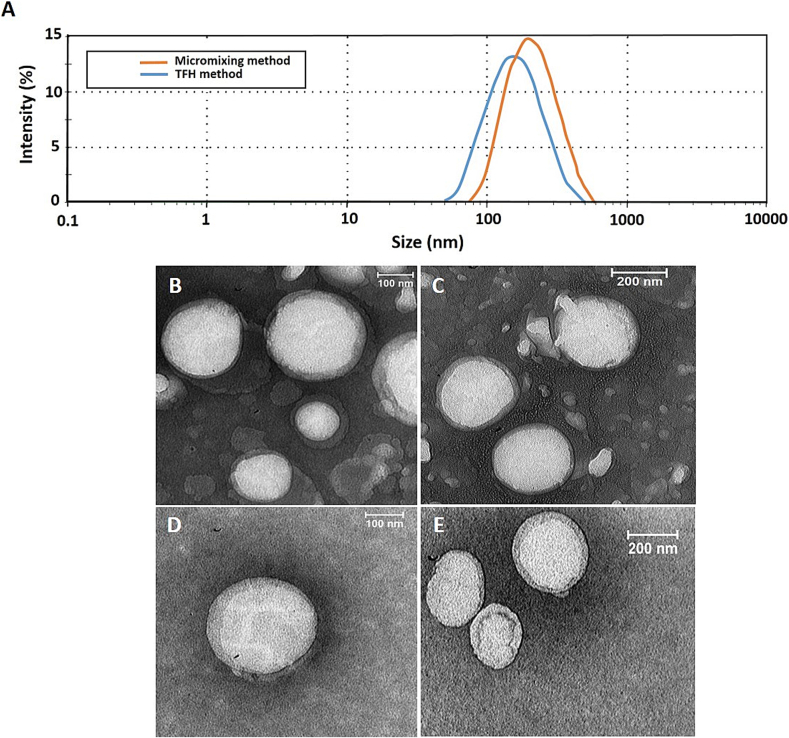
Diameter of nanoliposomes. Size of nanoparticles reported as intensity prepared by TFH method and coaxial micromixer (A), TEM images of empty liposomes prepared by both TFH (B and C) and micromixing (TFR = 150 μL/min and FRR = 2) (D and E) methods.

Fig. 5(A) shows the mean diameter of nanoparticles determined by intensity. PDI values were also less than 0.2, indicating the homogeneity of the nanoliposome population (Danaei et al., 2018). As expected, liposomes have a negative charge of −50 mV to −60 mV due to having phosphatidylserine (PS) in their formulations.

In agreement with this finding, previous studies reported that liposomes containing the same amount of negatively charged phospholipid (about 25 % to 30 % molar ratio) in different dispersant media including 5 % glucose (Klein et al., 2020), 20 mM HEPES buffer pH 7.4 (Rahnfeld et al., 2018), and 10 mM Tris, pH 7.4, 100 mM NaCl, 0.5 mM EDTA (Markones et al., 2018) had zeta potentials of −50 mV to −60 mV, −40 mV to −50 mV, and − 20 mV to −30 mV respectively. This difference in the negative charge values  is due to the difference in the ionic strength of the nanoparticle dispersant medium. Also, in another study, liposomes containing 25 % DOPS had high negative charge values, i.e. -50 mV to −60 mV (Weber et al., 2021).

The transmission electron microscopy (TEM) images show that the empty liposomes made by both TFH and micromixing methods have a spherical structure and their size is about 200 nm, which is similar to the DLS results Fig. 5(B-E).

The *EE%* of the hydrophobic ATC in the lipid bilayer of the nanoliposomes obtained by both methods was about 70 % (Table 4). The manufacturing method also affects the encapsulation efficiency. EE values can provide insight into whether a production method can be applicable in the industry (Correia et al., 2017; Kulkarni et al., 1995). The results prove that the micromixing procedure, benefiting from the synthesis of nanoliposomes and drug encapsulation in a continuous and one-step manner, enables obtaining a formulation with a more controlled structure that is comparable with the result of the film extrusion method.

In addition, to ensure that the micromixing method is cost-effective and that the lipid concentration remains constant at the ratio initially designed before nanoformulation production, it is important to verify the lipid recovery (Kastner et al., 2015). The obtained results revealed that at the same initial phospholipid content, the produced liposomal formulations using both methods, either empty or drug-loaded, have a similar amount of phospholipid concentration per vesicle (Table 5).

**Table 5:** Phosphate assay results of liposomal formulations after dialysis prepared by TFH (F) and micromixing ((M), TFR = 150 μL/min and FRR = 2) methods either empty (L(Empty)) or drug-loaded (L(ATC)). Data are denoted as mean ± S. D. (n = 3).

Method	Liposomal formulation	mM
TFH	L(Empty)_F_ (DOPC:DOPS:CHol) (35:25:40)	23.2 ± 1.4
	L(*ATC*)_F_ (DOPC:DOPS:CHol:ATC) (30:25:40:5)	18.75 ± 1.06
Micromixing	L(Empty)_M_ (DOPC:DOPS:CHol) (35:25:40)	26.7 ± 0.87
	L(*ATC*)_M_ (DOPC:DOPS:CHol:ATC) (30:25:40:5)	18.7 ± 0.65

#### 3.2.3 DSC thermograms of liposomal formulations

**Fig. 6:**
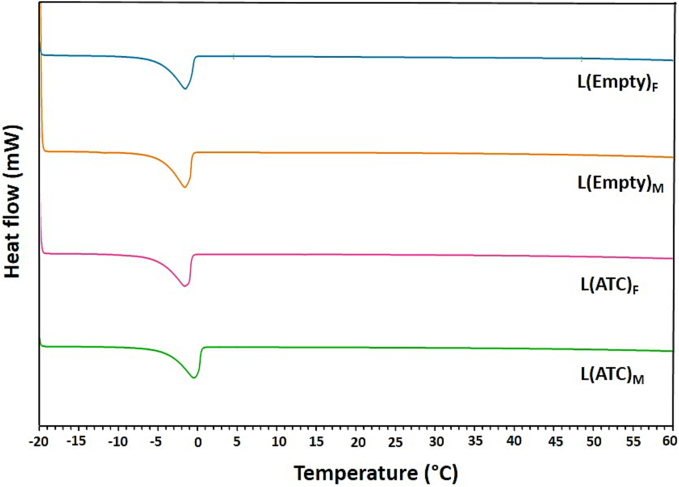
DSC curves of nanoliposomes. Thermograms of empty (L(Empty)) and ATC-containing (L(ATC)) liposomes prepared by both TFH (F) and micromixing ((M), TFR = 150 μL/min and FRR = 2) methods.

Fig. 6 demonstrates DSC thermograms related to nanoliposomal formulations prepared by two methods of TFH and micromixing. T_m_ of phospholipids is the phase transition temperature from gel-like solid phase to liquid crystalline phase. The T_m_ of DOPS and DOPC phospholipids is −11 °C and − 17 °C, respectively. Fig. 6 shows the endothermic peaks of empty and ATC-containing liposomes prepared by both methods, which are around −2 °C. Since we have only a peak, DSC thermograms indicate that the phospholipids are well mixed. Fig. 6 also indicates that the T_m_ of drug-containing liposomes is the same as empty liposomes and ATC as a hydrophobic drug is well incorporated into the phospholipid bilayer without any change in the T_m_ of liposomes. The presence of individual endothermic peaks of drug-loaded liposomes indicates that no interaction between the lipid components and the drug molecules has taken place (Rudra et al., 2010).

#### 3.2.4 Stability study of nanoliposomes

**Fig. 7:**
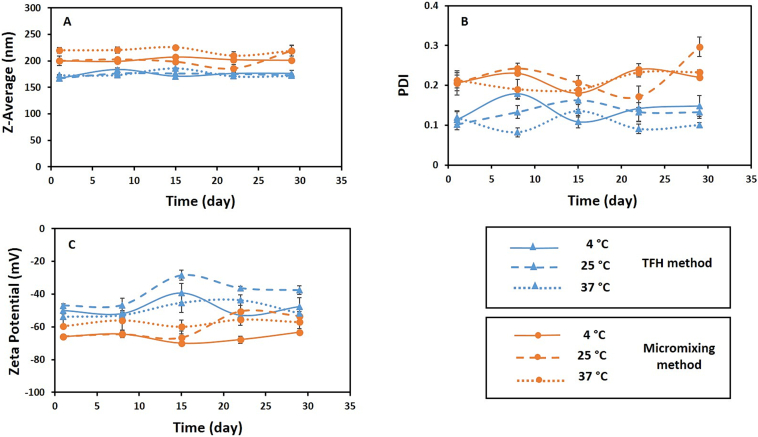
Stability study. The stability of empty liposomal formulations produced by TFH and micromixing (TFR = 150 μL/min and FRR = 2) methods during one month at 4 °C, 25 °C and 37 °C in terms of particle size (A), PDI (B) and zeta potential (C). Data are denoted as mean ± S·D, (*n* = 3). The difference in the stability of liposomes comparing three investigated temperatures is not significant for any of the methods (*P* > 0.05).

The physical instability of nanoliposomes occurs mainly due to aggregation, fusion and rupture of these nanoparticles in different time periods and temperatures. Generally, the physical stability of liposomal formulation is characterized based on two main features: particle size distribution and visual inspection (Jyothi et al., 2022; Thompson et al., 2006). No precipitation and turbidity were observed for the liposomes during one month at the three temperatures of 4 °C, 25 °C and 37 °C. The results showed that there was no significant difference (*P* > 0.05) in particle size, PDI and surface charge of liposomes in comparison between three temperatures across different time points (i.e. on days of 1, 8, 15, 22, and 29) for each of the TFH and micromixing methods, which indicates high stability of the formulations during this time period (Fig. 7). The stability of liposomes produced using both methods can be due to their high negative surface charge, which creates significant electrostatic repulsion and prevents the fusion and aggregation of nanoliposomes (Aghaei et al., 2021; Li et al., 2021). Also, the DLS results regarding the stability of liposomes prepared using TFH method and coaxial micromixer at three temperatures during one month are presented in Table S3 in the supplementary information. Additionally, TEM images of liposomes prepared by both methods after one month are included in the supplementary data (Fig. S3). As shown in Fig. S3(A-H), the size of liposomes prepared by both approaches did not change significantly after one month and it is in accordance with the results of DLS (Table S3).

#### 3.2.5 In vitro release profile of drug from nanoliposomes

**Fig. 8:**
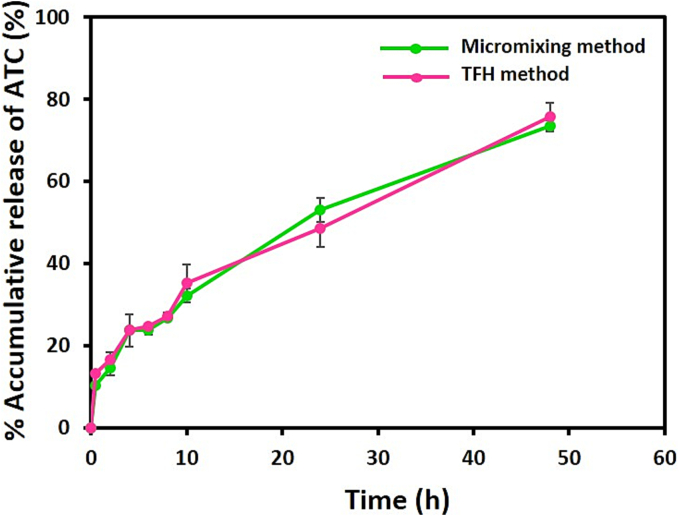
Comparison of release profiles. The in vitro release of ATC from liposomes produced by TFH and micromixing (TFR = 150 μL/min and FRR = 2) methods in Histidine/dextrose buffer pH (6.5) at 37 °C. Data are demonstrated as mean ± S·D, (*n* = 3). The difference in the release profile between the two methods is not significant (*P* > 0.05).

The coaxial micromixer was found to generate nanoliposomes with desirable characteristics similar to the liposomes produced by the TFH method. Fig. 8 shows that the ATC release profiles from liposomes prepared by both techniques in histidine/dextrose medium (10 mM, pH 6.5) and within 48 h at 37 °C do not have significant differences (P > 0.05). Drug release is mainly influenced by the physicochemical properties of liposomes such as size, lipid composition and lipid chain length, as well as where the drug molecule is placed within liposome structure and less affected by the manufacturing method (Correia et al., 2017; Kastner et al., 2015). The in vitro release profiles were explainable with some structural characteristics of the nanoliposomes. The burst release of ATC in the first hour is attributed to the presence of phospholipids with low Tm in the vesicle structure. This results in reduced payload retention and an accelerated rate of content release (Naeini et al., 2021). The drug release data of ATC-containing liposomes were fitted to several kinetic models such as zero-order, first-order, Hixson-Crowell, Korsmeyer-Peppas and Higuchi by the DD Solver program in Microsoft Excel. Most of these models are based on diffusion equations depending on the lipid composition of liposomes and release conditions (Jain and Jain, 2016). ATC release from nanoliposomes produced by both methods showed the best fit to the Higuchi model with a correlation coefficient close to 1 (R^2^ of 0.9919 and 0.9958 for ATC-loaded liposomes synthesized by TFH and micromixing methods, respectively). According to the Higuchi model, the mechanism of ATC release is diffusion. Previously, the Higuchi model has been successfully applied in studies to describe the release kinetics of hydrophobic drugs from nanoliposomes (Othman et al., 2022; Vu et al., 2020).

#### 3.2.6 Cytocompatibility comparison of liposomes

**Fig. 9:**
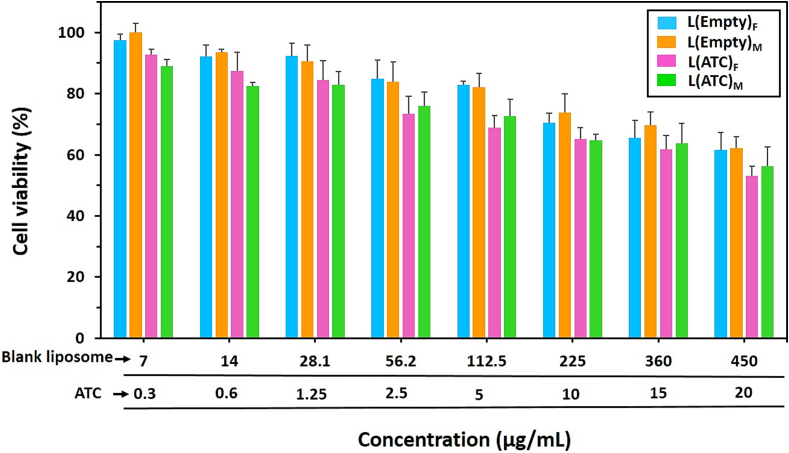
The cytocompatibility comparison of nanoliposomes. Viabilities of J774 macrophages cell line treated with nanoliposomes without drug (L(Empty)) or with drug (L(ATC)) at 37 °C for 48 h. Cell viability of empty and ATC-loaded liposomes prepared by both TFH (F) and micromixing ((M), TFR = 150 μL/min and FRR = 2) methods showed no significant differences in various concentrations (P > 0.05). Data are presented as mean ± S·D, (*n* = 3).

MTT test was performed to study the cytocompatibility of empty and ATC-containing liposomal formulations prepared by both TFH and micromixing methods. Fig. 9 shows that the viabilities of J774 cells treated with empty liposomes produced by both methods do not have significant differences in various concentrations (P > 0.05). Also, the treatment of macrophages with ATC-loaded liposomes made by both techniques demonstrated similar cell viability at different concentrations, consistent with a previous study that investigated cytotoxicity of liposomes prepared by TFH and microfluidic methods (Al-Amin et al., 2020). Within the tested concentrations range, the presence of ATC in drug-containing liposomes did not result in a significant change in cell viability compared to empty liposomes. Based on these findings, overall, as the concentration of liposome and ATC increased, a decreasing trend was observed for the viability of macrophages treated with empty and drug-containing liposomes, which is in agreement with the results of cell viability of blank and ATC-loaded liposomes in a previous study (Li et al., 2019). They demonstrated that at high concentration of ATC (10 μg/mL), the survival percentages of Human aortic endothelial cells (HAECs) treated with empty liposome and containing ATC were 70 % and 45 % respectively (Li et al., 2019), but in this study, the corresponding values are 70 % and 65 % respectively for macrophages. The lower toxicity of ATC-loaded liposomes in this study could be due to the high negative charge of these nanoparticles. In general, anionic liposomes have less cytotoxicity compared to cationic liposomes, which can be due to the similarity of negatively charged lipids used (such as phosphatidylserine (PS), phosphatidylglycerol (PG), and phosphatidic acid (PA)) in the synthesis of anionic liposomes with the lipid composition of natural cell membranes (Tseu and Kamaruzaman, 2023).

In previous study, the incorporation of anionic liposomes into cationic polyplexes has diminished their surface charge, thereby enhancing safety and lowering cytotoxicity of this nanosystem. (Kurosaki et al., 2014). In another study, adding an appropriate amount of PS as a negatively charged lipid to cationic liposomes has led to a decrease in the total charge of the liposome and, as a result, an increase in the safety of these carriers (Wang et al., 2023).

#### 3.2.7 In vitro cellular uptake of nanoliposomes

**Fig. 10:**
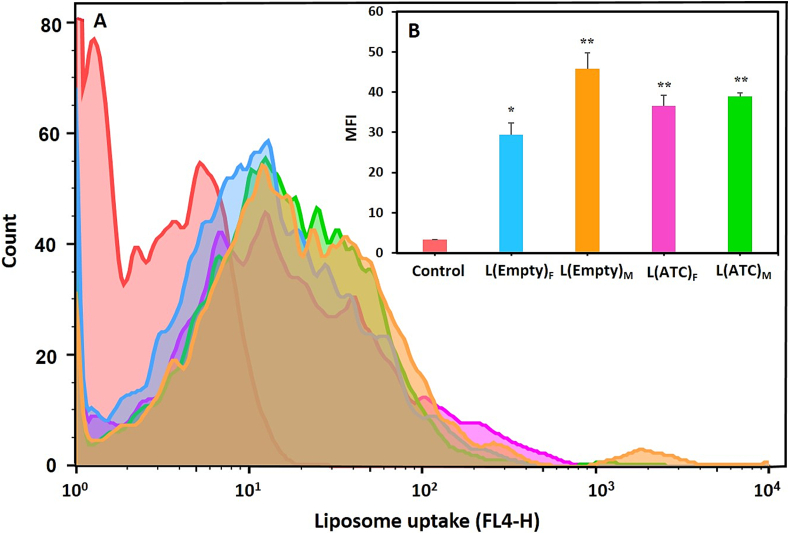
The in vitro cellular uptake of liposomes. A) Uptake of DiR-labeled nanoliposomes without drug (L(Empty)) or with drug (L(ATC)) by J774 macrophage cell line incubated at 37 °C for 4 h. The groups treated with liposomes prepared by TFH (F) and micromixing ((M), TFR = 150 μL/min and FRR = 2) methods compared to the control group (untreated macrophages) showed a significant level of cellular uptake. B) The mean fluorescence intensity (MFI) of different liposome uptake by the J774 cell line. Data are presented as mean ± S·D, (n = 3). Statistical differences in terms of significance are shown as (*: P < 0.05, **: P < 0.01).

The uptake of empty and ATC-containing nanoliposomes by J774 macrophage cells was evaluated using flow cytometry at 37 °C in laboratory conditions. Fig. 10 shows the macrophage cell line taken up the liposomes prepared using TFH method and coaxial micromixer. The mean fluorescence intensity (MFI) of the groups treated with DiR fluorescent dye-labeled liposomes, compared to the control group (i.e. untreated macrophages) indicated a significant difference (*P* < 0.01 and *P* < 0.05). These results suggested that the J774 cell line can efficiently uptake nanoliposomes in culture medium.

Various in vivo and in vitro studies have shown that the uptake of liposomes by macrophages depends on the different physicochemical properties of these nanoparticles such as size, surface charge and their composition (Dacoba et al., 2017; Getts et al., 2015). For example, positively or negatively charged nanoliposomes are more efficiently recognized and phagocytized by macrophages than neutral nanoparticles. Also, macrophages express a range of different classes of scavenger receptors on their surface, some of which are involved in the binding and uptake of liposomes containing negatively charged phospholipids, such as PS. On the other hand, incorporating PS as an apoptotic signal in the composition of the nanoparticles increases their uptake by phagocytes such as macrophages (Dangkoub et al., 2021; Matsumoto et al., 2017; Tempone et al., 2004).

Previously, a study showed that anionic liposomes containing PS (zeta potential: −66 mV) exhibited more rapidly and higher uptake by macrophages compared to their negatively charged nanoliposomes containing PG (zeta potential: −55 mV) and their neutral counterparts (zeta potential: −2 mV), this could be due to the presence of PS as a recognition signal for preferential phagocytosis by macrophage cells (Khadke et al., 2019).

In this study, the empty and drug-containing liposomes prepared by both methods were significantly taken up by the J774 macrophage cell line compared to the control group, which could be due to high negative charge (−55 to −65 mV) and the presence of phospholipid PS in these nanoliposomes. In addition, the difference observed in Fig. 10 may be attributed to the slightly larger size and slightly higher negative charge of empty and ATC-containing liposomes prepared by the micromixing method compared to those made by the TFH approach. Larger size and increased negative charge of nanoparticles can enhance their uptake by macrophages (Getts et al., 2015; Dangkoub et al., 2021).

## 4 Conclusion

This article reported a comparative study to investigate the capacity of a novel micromixer for the production of empty and drug-containing liposomes in comparison with the conventional TFH method. The obtained results revealed that both approaches produced empty and ATC-containing liposomes with similar physicochemical properties. Drug release profiles of the ATC-loaded liposomes prepared via both methods did not demonstrate significant differences. Analysis of stability and DSC thermograms showed good and similar results for liposomes made by both techniques. In addition, the cellular uptake and cytocompatibility of nanoliposomes produced using micromixing and TFH methods were evaluated on the J774 macrophage cell line, demonstrating similar results. The presented micromixer is cost-effective without specialized equipment, and compatible with various solvents. Compared to conventional techniques, microfluidics, i.e. micromixing, offers high production yield in a reproducible manner. Importantly, extrusion and sonication steps were eliminated from the micromixing-based production process that decreased the overall cost and time of operation, reducing the chance of batch-to-batch variations significantly.

In this way, optimization steps to finalize a nanoformulation could be carried out with minimized time and cost. Further assessment of physical and chemical properties and the cost of nanoliposomes produced by both micromixing and conventional methods could be of interest for some industrial considerations.

## CRediT authorship contribution statement

**Faezeh Dangkoub:** Writing – review & editing, Writing – original draft, Investigation, Formal analysis, Conceptualization. **Mehri Bemani Naeini:** Writing – original draft, Investigation. **Shima Akar:** Writing – review & editing, Writing – original draft, Formal analysis. **Ali Badiee:** Writing – review & editing, Validation, Supervision. **Mahmoud Reza Jaafari:** Methodology, Conceptualization. **Mojtaba Sankian:** Writing – review & editing, Software, Investigation. **Mohsen Tafaghodi:** Writing – review & editing, Validation, Resources, Funding acquisition. **Seyed Ali Mousavi Shaegh:** Writing – review & editing, Validation, Supervision, Resources, Methodology, Conceptualization.

## Declaration of competing interest

The authors declare that they are no conflicts of interest.

## Data Availability

Data will be made available on request.

## Appendix A Supplementary data

Supplementary material Image 1
