# Supplementary material for: Preparation of atorvastatin calcium-loaded liposomes using thin-film hydration and coaxial micromixing methods: A comparative study
Source: Int J Pharm X. 2024 Nov 29;8:100309. doi: 10.1016/j.ijpx.2024.100309 (PMC11653151; doi:10.1016/j.ijpx.2024.100309)
Supplement: Supplementary file 1 — Supplementary material [file mmc1.docx]

**Supplementary information**

**Preparation of atorvastatin calcium-loaded liposomes using thin-film hydration and coaxial micromixing methods: a comparative study**

Faezeh Dangkoub ^1,2,3^, Mehri Bemani Naeini ^1,2§^, Shima Akar^3,4§^, Ali Badiee ^1,2^, [Mahmoud Reza Jaafari](https://www.researchgate.net/lab/Mahmoud-Reza-Jaafari-Lab-mahmoud-reza-Jaafari?_sg=dpTHbFfYPpxSiPTesE3g1zUrGdZ2Y1CYHx-_ywkpfecHg8pqBeWmr6g9Elt8dj2DfR7337lDqdKNrerPVV6VIH1NAA) ^1,2,5^, Mojtaba Sankian ^6^, Mohsen Tafaghodi ^1,2^, Seyed Ali Mousavi Shaegh ^3,4,7*^

1. Department of Pharmaceutical Nanotechnology, School of Pharmacy, Mashhad University of Medical Sciences, Mashhad, Iran
2. Nanotechnology Research Center, Pharmaceutical Technology Institute, Mashhad University of Medical Sciences, Mashhad, Iran
3. Laboratory of Microfluidics and Medical Microsystems, Research Institute for Medical Sciences, Mashhad University of Medical Sciences, Mashhad, Iran
4. Orthopedic Research Center, Mashhad University of Medical Sciences, Mashhad, Iran
5. Biotechnology Research Center, Pharmaceutical Technology Institute, Mashhad University of Medical Sciences, Mashhad, Iran
6. Immunology Research Center, Mashhad University of Medical Sciences, Mashhad, Iran
7. Clinical Research Unit of Ghaem Hospital, Mashhad University of Medical Sciences, Mashhad, Iran

**Corresponding author*: Seyed Ali Mousavi Shaegh

*§ Equal contributions*

Clinical Research Unit, and Orthopedic Research Center, School of Medicine,

Room 37, 1st Floor, Narjes Building, Ghaem Hospital, Ahmadabad Ave.,

Laboratory of Microfluidics and Medical Microdevices, Research Institute for Medical Sciences,

Mashhad University of Medical Sciences, Mashhad, Iran

Tel: +98-5138417403 Email: [mousavisha@mums.ac.ir](mailto:Abnouskh@mums.ac.ir)

**Introduction**


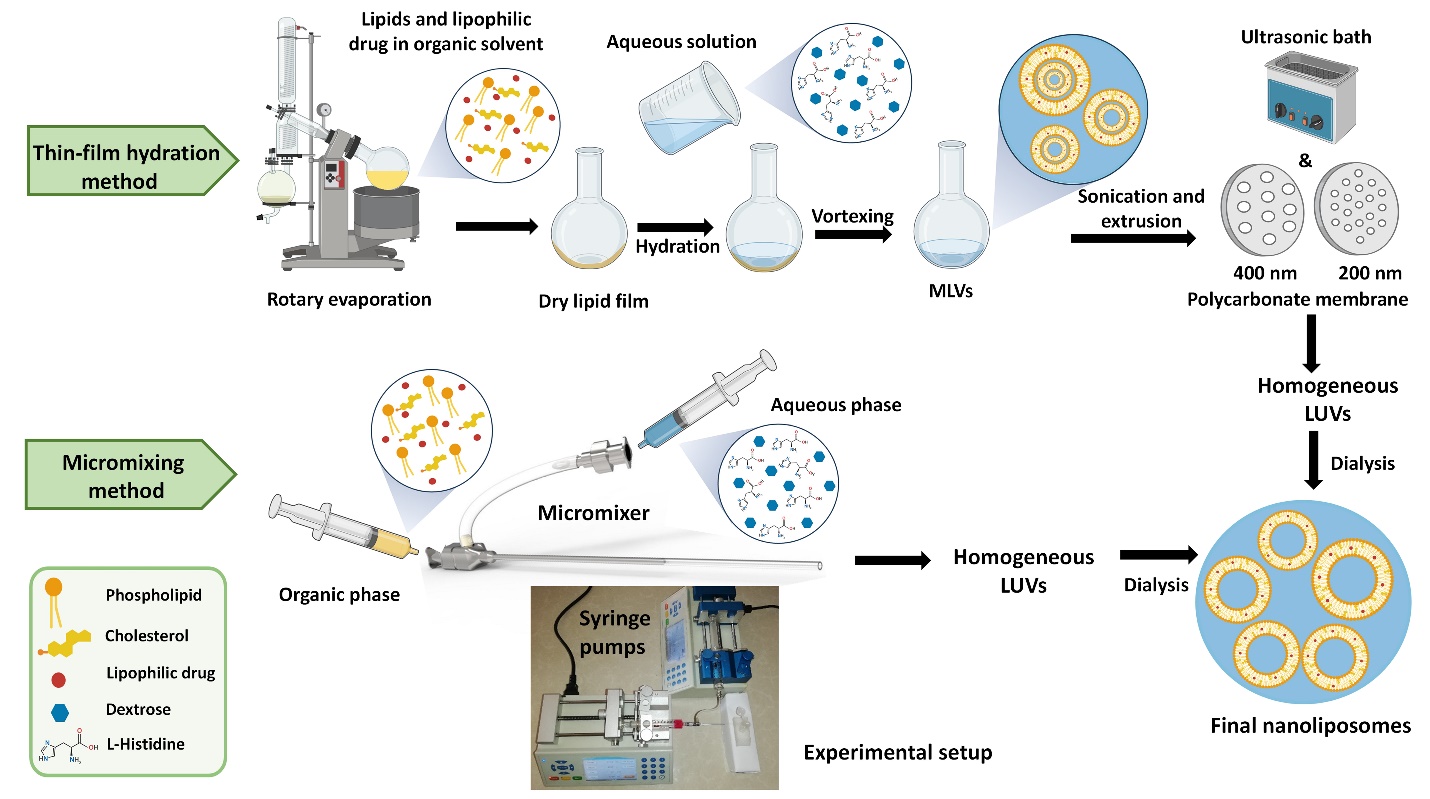


**Fig. S1**. Comparison of the TFH method with the one-step micromixing approach for the preparation of nanoliposomes.

**2. Materials and methods**

# **2.2. Numerical study**

## **2.2.1. Micromixers geometry and governing equations**

It was assumed that the fluid flow through the mixer had steady-state and three-dimensional (3D) conditions, and behaved as a Newtonian fluid with constant properties. With these assumptions, governing equations to determine the mixing behavior of the two streams were presented in the following forms [1]:

| $\frac{\boldsymbol{1}}{\boldsymbol{r}}\frac{\boldsymbol{\partial}}{\boldsymbol{\partial r}}\boldsymbol{(ru}$*(+* $\frac{\boldsymbol{\partial w}}{\boldsymbol{\partial z}}\boldsymbol{=0}$ | (1) |
| --- | --- |
| $\boldsymbol{\rho}\left( \boldsymbol{u}\frac{\boldsymbol{\partial u}}{\boldsymbol{\partial r}}\boldsymbol{+w}\frac{\boldsymbol{\partial u}}{\boldsymbol{\partial z}} \right)\boldsymbol{=-}\frac{\boldsymbol{\partial p}}{\boldsymbol{\partial r}}\boldsymbol{+\mu((}\frac{\boldsymbol{1}}{\boldsymbol{r}}\frac{\boldsymbol{\partial}}{\boldsymbol{\partial r}}\boldsymbol{(r}\frac{\boldsymbol{\partial u}}{\boldsymbol{\partial r}}$*))-* $\frac{\boldsymbol{u}}{\boldsymbol{r}^{\boldsymbol{2}}}\boldsymbol{+}\frac{\boldsymbol{\partial}^{\boldsymbol{2}}\boldsymbol{u}}{\boldsymbol{\partial}\boldsymbol{z}^{\boldsymbol{2}}}\boldsymbol{)}$ | (2) |
| $\boldsymbol{\rho}\left( \boldsymbol{u}\frac{\boldsymbol{\partial w}}{\boldsymbol{\partial r}}\boldsymbol{+w}\frac{\boldsymbol{\partial w}}{\boldsymbol{\partial z}} \right)\boldsymbol{=-}\frac{\boldsymbol{\partial p}}{\boldsymbol{\partial z}}\boldsymbol{+\mu((}\frac{\boldsymbol{1}}{\boldsymbol{r}}\frac{\boldsymbol{\partial}}{\boldsymbol{\partial r}}\boldsymbol{(r}\frac{\boldsymbol{\partial w}}{\boldsymbol{\partial r}}$*))*$\boldsymbol{+}\frac{\boldsymbol{\partial}^{\boldsymbol{2}}\boldsymbol{w}}{\boldsymbol{\partial}\boldsymbol{z}^{\boldsymbol{2}}}\boldsymbol{)}$ | (3) |
| $\boldsymbol{u}\frac{\boldsymbol{\partial C}}{\boldsymbol{\partial r}}\boldsymbol{+w}\frac{\boldsymbol{\partial C}}{\boldsymbol{\partial z}}\boldsymbol{=D(}\frac{\boldsymbol{1}}{\boldsymbol{r}}\frac{\boldsymbol{\partial}}{\boldsymbol{\partial r}}\left( \boldsymbol{r}\frac{\boldsymbol{\partial C}}{\boldsymbol{\partial r}} \right)\boldsymbol{+}\frac{\boldsymbol{\partial}^{\boldsymbol{2}}\boldsymbol{C}}{\boldsymbol{\partial}\boldsymbol{z}^{\boldsymbol{2}}}\boldsymbol{)}$ | (4) |

where $\boldsymbol{u}\left( \frac{\boldsymbol{m}}{\boldsymbol{s}} \right)\boldsymbol{,w}\left( \frac{\boldsymbol{m}}{\boldsymbol{s}} \right)\boldsymbol{, \rho}\left( \frac{\boldsymbol{kg}}{\boldsymbol{m}^{\boldsymbol{3}}} \right)\boldsymbol{, \mu}\left( \boldsymbol{Pa\cdot s} \right)$, $\boldsymbol{p}\left( \boldsymbol{Pa} \right)$, $\boldsymbol{C}\left( \frac{\boldsymbol{mol}}{\boldsymbol{m}^{\boldsymbol{3}}} \right)$ and $\boldsymbol{D(}\boldsymbol{m}^{\boldsymbol{2}}\boldsymbol{/s)}$ are the radial velocity, axial velocity, density, dynamic viscosity, pressure, species concentration, and diffusion coefficient, respectively.

Following is a description of the boundary conditions:

- THF enters inlet 1 under the following conditions:

| $\boldsymbol{u=}\boldsymbol{Q}_{\boldsymbol{T}}\boldsymbol{/}\boldsymbol{A}_{\boldsymbol{T}}\boldsymbol{, , w=0, C=1}$ | (5) |
| --- | --- |

where $Q_{T}$ and $A_{T}$ are, in order, THF flow rate and internal cross-sectional area of the coaxial micromixer, respectively.

- The Deionized water enters inlet 2 under the succeeding conditions:

| $\boldsymbol{u=}\boldsymbol{Q}_{\boldsymbol{w}}\boldsymbol{/}\boldsymbol{A}_{\boldsymbol{w}}\boldsymbol{,w=0, C=0}$ | (6) |
| --- | --- |

where $Q_{w}$ and $A_{w}$ are flow rate and external cross-sectional area of the coaxial micromixer, respectively.

- Mixer outlet has pressure outlet condition where static gauge pressure is zero.
- For the micromixer walls, except the axis shown in **Fig. 2,** no-slip boundary condition (zero velocity) is applied.
- Axis boundary condition

| $\frac{\boldsymbol{\partial p}}{\boldsymbol{\partial\theta}}\boldsymbol{=}\frac{\boldsymbol{\partial}\boldsymbol{v}_{\boldsymbol{r}}}{\boldsymbol{\partial\theta}}\boldsymbol{=}\frac{\boldsymbol{\partial}\boldsymbol{v}_{\boldsymbol{z}}}{\boldsymbol{\partial\theta}}\boldsymbol{=0}$ | (7) |
| --- | --- |

The mixing efficiency (*MI*) of the species can be calculated by the following equations [2]:

| $\sigma=\sqrt{\frac{1}{M}\sum_{i=1}^{M} {{(c}_{i}-\bar{c)}}^{2}}$ | (8) |
| --- | --- |
| $\bar{c}=\frac{\sum_{1}^{m} c_{i}}{M}$ | (9) |
| $MI=1-\sqrt{\frac{\sigma^{2}}{{\sigma_{max}}^{2}}}$ | (10) |

In Eqs. (8) and (10),$\sigma_{\max}, \sigma, c_{i}$and $M$represent the maximum variance of the mixture, standard deviation, the mass fraction of node *i,* and the quantity of nodes inside the cross-section area, respectively*.* $\bar{c}$ refers to the mass fraction in which the mixing efficiency is 100% (equal to 0.25 in case the flow rate ratio is 3), and $\eta$represents the mixing efficiency between 0 to 1, indicating totally segregated phase and completely mixed phase, respectively.

[1] J. Lopez, J. Shen, An efficient spectral-projection method for the Navier–Stokes equations in cylindrical geometries: I. Axisymmetric cases, Journal of Computational Physics, 139 (1998) 308-326.

[2] S. Akar, A. Taheri, R. Bazaz, E. Warkiani, M. Shaegh, Twisted architecture for enhancement of passive micromixing in a wide range of Reynolds numbers, Chemical Engineering and Processing-Process Intensification, 160 (2021) 108251.

**2.2.2. Numerical solution procedure**

**Table S1**

The effects of grid number on mixing efficiency at FRR and TFR of 2 and 150 μL/min, respectively.

| **Mesh size** | **Mixing Efficiency (%)** | **Error (%)** |
| --- | --- | --- |
| 182,000 | 0.936 |  |
| 365,000 | 0.959 | 2.40405 |
| **735,000** | **0.974** | **1.580171** |
| 1,420,000 | 0.97 | 0.426404 |

**3. Results and discussion**

**3.2. Experimental results**

**3.2.1. DOE study to investigate the effects of micromixing inputs on liposome characteristics**

**3.2.1.1. Analysis**

**ANOVA table for the fitting model**

**Table S2** presents the ANOVA data for a three-factor model assessing size, PDI, and zeta potential based on the input parameters. The analysis indicates that most of the inputs significantly impact the size of the liposomes. However, for zeta potential and PDI, only some parameters exhibit a significant effect, while others do not significantly influence the results. Consequently, certain inputs show an insignificant impact on the responses for zeta potential and PDI. Based on this model, predictive equations (Equations 11, 12, and 13) have been developed for each response variable: size, PDI, and zeta potential, reflecting their respective relationships with the input parameters.

| $\sqrt{\mathbf{size}}\mathbf{= 18.68 - 0.9831A + 2.53B + 1.89C - 0.7548AB + 0.5159AC + 2.08BC - 6.79}\mathbf{A}^{\mathbf{2}}\mathbf{- 0.5725}\mathbf{B}^{\mathbf{2}}\mathbf{- 1.70}\mathbf{C}^{\mathbf{2}}\mathbf{- 3.04}\mathbf{A}^{\mathbf{2}}\mathbf{B- 0.9241}\mathbf{A}^{\mathbf{2}}\mathbf{C}$ | (11) |
| --- | --- |

| $\frac{\mathbf{1}}{\mathbf{PDI}}\mathbf{= 4.4560 - 2.27A - 0.6971B + 0.5779C + 0.7538AB - 0.3237AC - 0.3563BC + 2}\mathbf{A}^{\mathbf{2}}\mathbf{+ 0.0494}\mathbf{B}^{\mathbf{2}}\mathbf{- 0.4670}\mathbf{C}^{\mathbf{2}}$ | (12) |
| --- | --- |

| $\mathbf{Zeta potential=-56.92 - 1.5458A - 4.9417B - 8.3125C - 1.825AB - 7.15AC - 0.3917BC + 19.5183}\mathbf{A}^{\mathbf{2}}\mathbf{+ 6.5433}\mathbf{B}^{\mathbf{2}}\mathbf{- 5.2483}\mathbf{C}^{\mathbf{2}}$ | (13) |
| --- | --- |

The analysis of the R², adjusted R², and predicted R² values reveals important insights into the performance of the models used for the responses. In the first scenario, the R² value of 0.97 indicates that 97% of the variability in the response variable is explained by the model, demonstrating an excellent fit. The adjusted R², also at 0.97, confirms the robustness of the model while accounting for the number of predictors, suggesting that the model is well-specified without overfitting. Additionally, a predicted R² of 0.95 indicates strong predictive capability, as it reflects the model's ability to accurately forecast outcomes on new data. In contrast, the second scenario shows an R² of 0.86, which signifies that 86% of the variability is explained, still indicating a good fit but less optimal than the first model. The adjusted R² of 0.82 suggests that some predictors may not significantly contribute to explaining the response variability, and the predicted R² of 0.73 indicates a decline in the model's predictive accuracy when applied to unseen data. Overall, while the first model demonstrates exceptional fit and predictability, the second model, although acceptable, may benefit from further refinement to enhance its predictive performance.

**Table S2** The ANOVA table for three fitting model

|  | Size | | | PDI | | zeta potential | |
| --- | --- | --- | --- | --- | --- | --- | --- |
| Parameter | **p-value** | **Significance**  **(p < 0.05)** | | **p-value** | **Significance**  **(p < 0.05)** | **p-value** | **Significance**  **(p < 0.05)** |
| A: FRR | < 0.0001 | Significant | | < 0.0001 | Significant | 0.2021 | Insignificant |
| B: TFR | < 0.0001 | Significant | | 0.0007 | Significant | 0.0002 | Significant |
| C: LC | < 0.0001 | Significant | | 0.0039 | Significant | < 0.0001 | Significant |
| AB | 0.0006 | Significant | | 0.0073 | Significant | 0.2854 | Insignificant |
| AC | 0.0150 | Significant | | 0.2314 | Insignificant | 0.0001 | Significant |
| BC | < 0.0001 | Significant | | 0.1887 | Insignificant | 0.8174 | Insignificant |
| A² | < 0.0001 | Significant | | < 0.0001 | Significant | < 0.0001 | Significant |
| B² | 0.0062 | Significant | | 0.8500 | Insignificant | 0.0003 | Significant |
| C² | < 0.0001 | Significant | | 0.0797 | Insignificant | 0.0028 | Significant |
| A²B | < 0.0001 | Significant | | NA |  | NA |  |
| A²C | 0.0026 | Significant | | NA |  | NA |  |
| R² | |  | 0.9795 |  | 0.8608 |  | 0.8672 |
| Adjusted R² | |  | 0.9734 |  | 0.8287 |  | 0.8366 |
| Predicted R²  Adequate precision | |  | 0.9570  42.7683 |  | 0.7319  17.8950 |  | 0.7591  16.3569 |
| Fitted equation | |  | Eq. 11 |  | Eq. 12 |  | Eq. 13 |

**Fig. S2.** illustrates the actual versus predicted results for size, PDI, and zeta potential, demonstrating a strong linear relationship among the three response variables. The data points are closely distributed around the linear regression line, indicating that the model effectively captures the underlying trends in the data. This linearity suggests that the predictive model is reliable and accurate, as the predicted values closely match the actual measurements. The tight clustering of the points around the line reinforces the model's capability to forecast outcomes consistently across different conditions, further validating its effectiveness for predicting liposome characteristics based on the input parameters.

**Fig. S2.** The actual versus predicted results for (A) size, (B) PDI and (C) zeta potential

**3. Results and discussion**

**3.2.4. Effect of manufacturing method on stability**

**Table S3**

Stability results of empty liposomal formulations produced by TFH (A) and micromixing ((B) TFR = 150 μL/min and FRR = 2) methods during one month at 4 °C, 25 °C and 37 °C in terms of particle size (A), PDI (B) and zeta potential (C). Data are denoted as mean ± S.D, (n = 3). The difference in the stability of liposomes comparing the three investigated temperatures is not significant for any of the methods (P>0.05).

| **A 4 °C** | | | | **25 °C** | | | **37 °C** | | |
| --- | --- | --- | --- | --- | --- | --- | --- | --- | --- |
| **Day** | **Z-Average (nm)** | **PDI** | **Zeta Potential (mV)** | **Z-Average (nm)** | **PDI** | **Zeta Potential (mV)** | **Z-Average (nm)** | **PDI** | **Zeta Potential (mV)** |
| **1** | 169.1±3.1 | 0.114±0.02 | -50±2.1 | 167±4.9 | 0.101±0.01 | -47.5±1.1 | 173±1.2 | 0.119±0.016 | -53.9±4.9 |
| **8** | 183.5±3.3 | 0.179±0.01 | -51.8±4.3 | 176.5±3.8 | 0.133±0.04 | -46.7±4.1 | 173.6±2.6 | 0.082±0.011 | -52.9±1.3 |
| **15** | 170.7±4.1 | 0.108±0.01 | -39.3±5.8 | 176.6±5.8 | 0.163±0.03 | -28.3±2.9 | 186.1±0.28 | 0.136±0.015 | -45.3±5.8 |
| **22** | 176±4.2 | 0.142±0.03 | -52.8±2.4 | 175.7±2.5 | 0.133±0.02 | -36.2±1.1 | 171.1±1.1 | 0.09±0.012 | -43.6±3.2 |
| **29** | 176.2±5.8 | 0.148±0.02 | -47.6±5.3 | 173.2±2.05 | 0.132±0.01 | -37.5±2.7 | \| 171.9±2.1 \| \| --- \| | 0.1±0.005 | -51±1.2 |

| **B 4 °C** | | | | **25 °C** | | | **37 °C** | | |
| --- | --- | --- | --- | --- | --- | --- | --- | --- | --- |
| **Day** | **Z-Average (nm)** | **PDI** | **Zeta Potential (mV)** | **Z-Average (nm)** | **PDI** | **Zeta Potential (mV)** | **Z-Average (nm)** | **PDI** | **Zeta Potential (mV)** |
| **1** | 200.2±9 | 0.206±0.03 | -65±1.4 | 200±3.2 | 0.206±0.019 | -65±1.4 | 220±5.4 | 0.213±0.019 | -59.6±0.8 |
| **8** | 199.4±1.1 | 0.232±0.004 | -64.4±1.1 | 202.8±1 | 0.242±0.013 | -64.6±1.8 | 220.4±5.4 | 0.19±0.025 | -56±5.9 |
| **15** | 207.3±4.3 | 0.182±0.021 | -69.2±0.78 | 198.5±4.1 | 0.206±0.017 | -66.7±4.3 | 225.7±0.7 | 0.189±0.007 | -60±4.2 |
| **22** | 202.3±5.8 | 0.242±0.015 | -67.8±2.1 | 185.4±7.9 | 0.171±0.027 | -56.6±3.8 | 209.7±7.5 | 0.232±0.01 | -55.5±3.6 |
| **29** | 201.5±0.35 | 0.227±0.002 | -63.3±0.14 | 219.3±10.2 | 0.296±0.024 | -53.5±0.5 | 224.4±10 | 0.232±0.006 | -57.1±4 |

As shown in the **Table S3**, the changes in the characteristics of liposomes in terms of size, PDI, and zeta potential between three temperatures of 4 **°C,** 25 **°C** and 37 **°C** during one month for each of the TFH and micromixing methods are not significant.


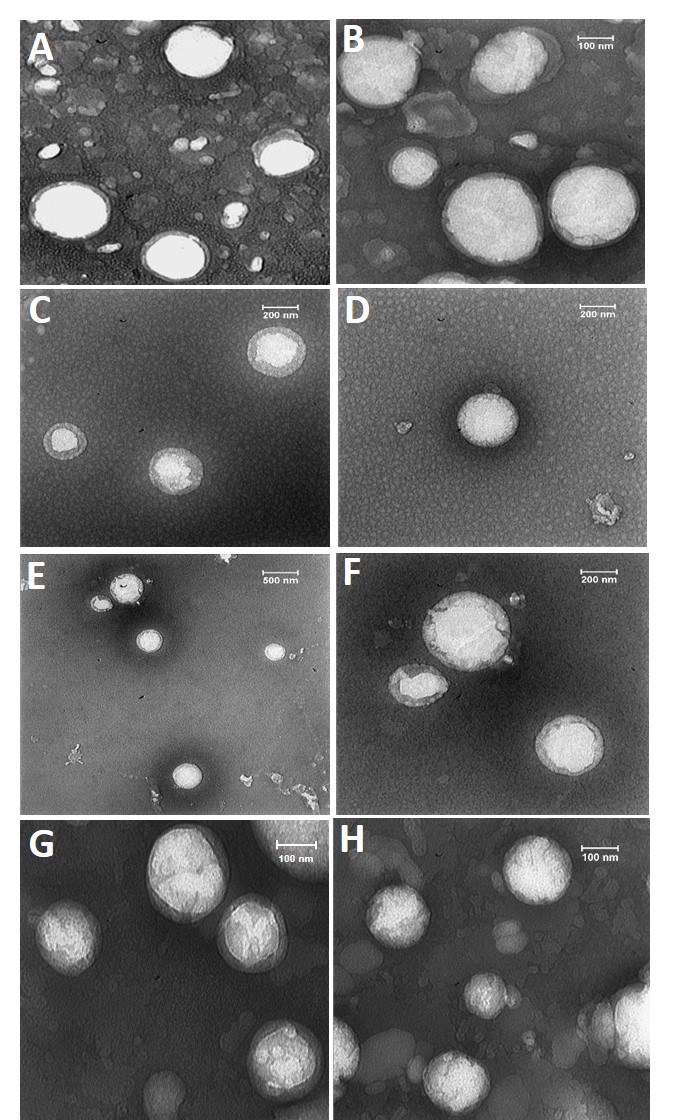


**Fig. S3**. TEM images of empty liposomes prepared by both TFH (A and B are at 4 °C and 25 °C respectively) and micromixing (TFR = 150 μL/min and FRR = 2) (C and D are at 4 °C and E and F are at 25 °C) methods after one month.

As shown in **Fig. S3** (**A-H**), the size of liposomes produced by two methods did not change significantly after one month that is consistent with the results of DLS (**Table S3**).
